# Predicting and comparing the long-term impact of lifestyle interventions on individuals with eating disorders in active population: a machine learning evaluation

**DOI:** 10.3389/fnut.2024.1390751

**Published:** 2024-08-07

**Authors:** Khadijeh Irandoust, Kamdin Parsakia, Ali Estifa, Gholamreza Zoormand, Beat Knechtle, Thomas Rosemann, Katja Weiss, Morteza Taheri

**Affiliations:** ^1^Department of Sport Sciences, Imam Khomeini International University, Qazvin, Iran; ^2^Department of Psychology and Counseling, KMAN Research Institute, Richmond Hill, ON, Canada; ^3^Department of Physical Education, Huanggang Normal University, Huanggang, China; ^4^Medbase St. Gallen Am Vadianplatz, St. Gallen, Switzerland; ^5^Institute of Primary Care, University of Zürich, Zürich, Switzerland; ^6^Department of Cognitive and Behavioural Sciences in Sport, Faculty of Sport Science and Health, University of Tehran, Tehran, Iran

**Keywords:** lifestyle interventions, long-term health outcomes, machine learning, prediction, eating disorders

## Abstract

**Objective:**

This study aims to evaluate and predict the long-term effectiveness of five lifestyle interventions for individuals with eating disorders using machine learning techniques.

**Methods:**

This study, conducted at Dr. Irandoust’s Health Center at Qazvin from August 2021 to August 2023, aimed to evaluate the effects of five lifestyle interventions on individuals with eating disorders, initially diagnosed using The Eating Disorder Diagnostic Scale (EDDS). The interventions were: (1) Counseling, exercise, and dietary regime, (2) Aerobic exercises with dietary regime, (3) Walking and dietary regime, (4) Exercise with a flexible diet, and (5) Exercises through online programs and applications. Out of 955 enrolled participants, 706 completed the study, which measured Body Fat Percentage (BFP), Waist-Hip Ratio (WHR), Fasting Blood Sugar (FBS), Low-Density Lipoprotein (LDL) Cholesterol, Total Cholesterol (CHO), Weight, and Triglycerides (TG) at baseline, during, and at the end of the intervention. Random Forest and Gradient Boosting Regressors, following feature engineering, were used to analyze the data, focusing on the interventions’ long-term effectiveness on health outcomes related to eating disorders.

**Results:**

Feature engineering with Random Forest and Gradient Boosting Regressors, respectively, reached an accuracy of 85 and 89%, then 89 and 90% after dataset balancing. The interventions were ranked based on predicted effectiveness: counseling with exercise and dietary regime, aerobic exercises with dietary regime, walking with dietary regime, exercise with a flexible diet, and exercises through online programs.

**Conclusion:**

The results show that Machine Learning (ML) models effectively predicted the long-term effectiveness of lifestyle interventions. The current study suggests a significant potential for tailored health strategies. This emphasizes the most effective interventions for individuals with eating disorders. According to the results, it can also be suggested to expand demographics and geographic locations of participants, longer study duration, exploring advanced machine learning techniques, and including psychological and social adherence factors. Ultimately, these results can guide healthcare providers and policymakers in creating targeted lifestyle intervention strategies, emphasizing personalized health plans, and leveraging machine learning for predictive healthcare solutions.

## 1 Introduction

Lifestyle interventions have been widely acknowledged for their significant role in improving health outcomes and tackling various health conditions (1). These interventions include a range of strategies like dietary regimes, exercise programs, counseling, and behavioral therapy which are key strategies in promoting healthy lifestyles and reducing the incidence of chronic diseases (2–5). However, assessing their long-term effectiveness is challenging because short-term outcomes may not always reflect their prolonged impact on health (6). This highlights the need for innovative methods to ensure these interventions’ lasting benefits. Machine learning is emerging as a valuable tool in this area, enabling the prediction and prioritization of the long-term effectiveness of lifestyle interventions based on periodically collected short-term data (7). Moreover, the specificity of eating disorders—ranging from anorexia nervosa and bulimia to binge-eating disorder—necessitates a highly personalized approach to treatment (3, 4), underlining the importance of tailoring interventions to meet the unique needs of each individual (8, 9). Studies have shown that factors such as low carbohydrate diets, dietary restraint, and specific eating behaviors are intricately linked to the management and prognosis of eating disorders (10–12). Additionally, the role of psychological factors, including personality traits like harm avoidance and novelty seeking, as well as the presence of co-occurring conditions such as borderline personality disorder, significantly influence treatment planning and effectiveness (13–16).

The study investigates five different lifestyle interventions, each with its own unique focus and methodology. One examines the combined effects of a walking regimen and dietary changes (17), while another looks at gym-based aerobic exercises combined with a dietary regime, focusing on health and body composition (17). The third uses digital platforms for exercise delivery, the fourth integrates counseling with exercise and diet, offering a broader approach to lifestyle changes, and the fifth combines exercise with a flexible diet, highlighting the value of personalized routines and dietary adaptability (18).

Various studies back the effectiveness of these interventions. Dietary interventions have been shown to significantly encourage healthy eating across different populations (19, 20). Interventions targeting walking behaviors are linked to weight loss and improved walking ability (21–24). Research on aerobic exercises combined with dietary regimes shows benefits for neurocognition (25), cardiovascular risk factors (26, 27), and metabolic effects in chronic conditions (28). Online exercise programs have proven effective in various situations, including during the COVID-19 pandemic (29–33).

Studies emphasizing personalized approaches in the integration of counseling, exercise, and dietary regimes (34–40) highlight the effectiveness of a flexible approach to exercise and diet, adaptable to individual needs and preferences (32, 41–45). The literature underscores the critical role of personalization in treatment plans, advocating for a holistic approach that considers the individual’s specific type of eating disorder, stage, co-occurring conditions, family dynamics, and psychological factors (46, 47). In this context, the potential of machine learning and genomics-driven approaches to personalize interventions offers promising avenues for enhancing treatment efficacy and patient outcomes in eating disorders (8). By leveraging these innovative technologies, healthcare providers can develop more nuanced and effective strategies tailored to the complex needs of individuals with eating disorders, ultimately contributing to better long-term health outcomes and quality of life.

In conclusion, this research endeavors to predict comparative effectiveness of various lifestyle interventions for people with eating disorders, aiming to contribute toward evidence-based recommendations for both public health strategies and individual health planning. By employing machine learning in this analysis, it could significantly influence the distribution of healthcare resources. Moreover, it assists in guiding people toward effective health strategies that could enhance their overall performance and quality of life.

## 2 Materials and methods

### 2.1 Study design and participants

The study carried out at the Dr. Irandoust’s Health Center at Qazvin was structured to investigate the impact of five different lifestyle interventions on individuals diagnosed with eating disorders, utilizing The Eating Disorder Diagnostic Scale [EDDS; (48)] solely for the initial diagnosis. The interventions included: (1) Counseling, exercise, and dietary regime, (2) Aerobic exercises with dietary regime, (3) Walking and dietary regime, (4) Exercise with a flexible diet, and (5) Exercises through online programs and applications. Initially, 955 participants were enrolled based on their EDDS diagnosis, with a final analytical sample of 706 participants after data cleaning.

To ensure the integrity and validity of the study outcomes, participants were randomly assigned to one of the five intervention groups using a stratified block randomization technique. This method was chosen to balance the distribution of participant characteristics across groups, such as age, gender, and initial severity of eating disorders, thereby reducing potential bias. Randomization was conducted using a computer-generated random numbers table, which assigned participants to the groups with equal probabilities. The randomization process was overseen by a statistician who was not involved in the data collection or analysis phases of the study.

Blinding was implemented during the assessment phases to minimize bias in the evaluation of outcomes. Assessors who conducted the follow-up measurements were blinded to the group assignments of participants. This was achieved by using coded identifiers rather than participant names or any identifiable information during the collection and recording of data. Furthermore, the data analysts were also blinded to the allocation during the statistical analysis phase. By maintaining this level of blinding, we aimed to ensure that the outcomes assessed were not influenced by the assessors’ or analysts’ knowledge of the participants’ group assignments, thus adhering to the principles of objective scientific measurement.

This longitudinal study, from August 2021 to August 2023, measured several health-related variables at four baselines including three-month, six-month, nine-month, and one-year intervals, and at the study’s conclusion after 2 years. The measured variables were: Body Fat Percentage (BFP), Waist-Hip Ratio (WHR), Fasting Blood Sugar (FBS), Low-Density Lipoprotein (LDL) Cholesterol, Total Cholesterol (CHO), Weight, and Triglycerides (TG). In our study, the selection of demographic and measurement variables was strategically driven by their relevance to the outcomes of eating disorder interventions and the robustness of predictive modeling. Demographic variables such as age, gender, education level, and annual income were included to account for socio-economic and biological factors that could significantly influence the efficacy of lifestyle interventions. Measurement variables like Body Fat Percentage (BFP), Waist-Hip Ratio (WHR), and others were chosen due to their direct relevance to health outcomes associated with eating disorders.

In sum, after participants were randomly assigned to one of the five intervention groups. Each group’s intervention was carefully monitored and consistently applied throughout the study. The interventions aimed to explore the effectiveness of different combinations of physical activity, dietary modifications, and psychological support on improving the health outcomes of individuals with eating disorders.

### 2.2 Measurements and tools

The study focused on a comprehensive set of health-related variables, including:

Eating disorders: the Eating Disorder Diagnostic Scale (EDDS), developed by Stice et al. (48), is a concise self-report tool designed to diagnose Anorexia Nervosa, Bulimia Nervosa, and Eating Disorder Not Otherwise Specified (EDNOS), accommodating changes in the DSM-5 to Other Specified Feeding or Eating Disorder (OSFED). Comprising 22 items that assess eating disorder symptoms and behaviors over the past 3–6 months, the EDDS evaluates concerns with eating, weight, and shape, binge eating episodes, and compensatory behaviors. It employs a scoring algorithm based on DSM-IV criteria, adaptable to DSM-5, to categorize individuals into diagnostic groups (49). Khabir et al. (50) confirmed its high internal consistency, test–retest reliability, and construct validity in Iranian population (50). To enhance the accuracy of our diagnoses and to address the complex nature of eating disorders, which can vary widely in their manifestations, we supplemented the Eating Disorder Diagnostic Scale (EDDS) assessments with face-to-face interviews for a subset of participants. These interviews were conducted by experienced psychiatrists specializing in eating disorders, using a structured clinical interview format that aligns with DSM-5 criteria. This approach allowed us to capture nuanced aspects of the disorders that self-reported measures alone might miss, such as subtle signs of distress, non-verbal cues, and inconsistencies in self-report versus observed behaviors.

Body fat percentage (BFP): a critical measure indicating the proportion of fat in the body, relevant to overall health and risk of diseases. This was measured using bioelectrical impedance analysis, which estimates body composition based on the resistance to an electrical current passed through the body (51).

Waist-hip ratio (WHR): this metric, calculated as the waist circumference divided by hip circumference, serves as an indicator of fat distribution and potential health risks. Measurements of waist and hip circumferences were taken using a standard measuring tape, ensuring accuracy and consistency in the measurement technique (51).

Fasting blood sugar (FBS): essential for assessing glucose metabolism and identifying risks for diabetes. Participants were instructed to fast for at least 8 h prior to testing. Blood samples were then collected and analyzed using standard laboratory techniques (52).

Low-density lipoprotein (LDL) cholesterol: often referred to as ‘bad’ cholesterol, higher levels of which are associated with an increased risk of cardiovascular diseases. Blood samples were used for lipid profiling, including the measurement of LDL cholesterol, using enzymatic assays in a certified laboratory (52).

Triglycerides (TG): a type of fat found in the blood, where high levels can increase the risk of heart disease. This measurement was also derived from the blood samples, using enzymatic analysis to determine triglyceride levels (52).

Cholesterol (CHO): overall cholesterol levels, including LDL and HDL, provide insights into heart disease risk. Total cholesterol levels, including both LDL and HDL, were assessed through blood tests, following standardized laboratory procedures (52).

Weight and height: the heights and weights of the subjects were measured with a precision of 0.1 kg using a SECA model 720 scale manufactured in Germany, and a SECA model 220 stadiometer, also made in Germany, with a precision of 0.1 m.

For the assessment of body composition, a Korean-made model 720 body composition measurement device was utilized. Venous blood sampling from the antecubital vein was conducted after a 12-h fasting period at all experimental stages, and the samples were analyzed using an enzymatic colorimetric method (GPO – PAP) for single-point measurement through a photometric approach with kits supplied by Pars Azmoon company.

### 2.3 Interventions

#### 2.3.1 Counseling, exercise, and dietary regime

Initially, participants were introduced to the program through one-on-one counseling sessions. These sessions aimed to establish a rapport, understand individual health goals, and identify any psychological barriers to change. As suggested by previous studies (53). Counselors used motivational interviewing techniques to encourage participants to articulate their health aspirations and the personal significance of achieving these goals. Simultaneously, as the researchers advised (54, 55) a personalized exercise regimen was developed for each participant, considering their current fitness level, preferences, and any physical constraints. This regime started with low to moderate-intensity activities to build a habit of regular physical activity without causing burnout or injury. Over time, as participants grew more comfortable and capable, the intensity and variety of exercises were gradually increased. This careful calibration ensured that the exercise component remained challenging yet achievable. Dietary guidance was another cornerstone of the intervention. Registered dietitians crafted tailored eating plans that balanced nutritional needs with personal tastes and lifestyle factors. These plans were not rigid diets but flexible frameworks designed to foster healthy eating habits. Participants learned about portion control, nutrient density, and how to make healthier food choices in real-world scenarios. This educational aspect was crucial for empowering participants to make informed decisions about their nutrition outside the structured environment of the intervention. Ultimately, regular follow-up sessions allowed for the adjustment of plans based on progress and feedback, ensuring that the intervention remained responsive to each participant’s evolving needs.

#### 2.3.2 Aerobic exercises with dietary regime

As suggested by Charatcharoenwitthaya et al. (56) and Narayan (57), the intervention started with moderate-intensity aerobic exercises, gradually increasing in intensity and duration based on individual progress and tolerance. Throughout the study, participants were guided by fitness experts to engage in various aerobic exercises such as brisk walking, jogging, cycling, and swimming, tailored to their preferences and capabilities. Concurrently, according to Cowell’s (58) and Galdino et al.’s (59) recommendations, dietitians provided personalized dietary plans that complemented the physical activities, focusing on balanced intake of macronutrients and sufficient hydration. This combination aimed to optimize the health benefits of aerobic exercise by supporting muscle recovery and energy levels. Regular assessments were conducted to monitor health improvements and adjust exercise and dietary plans accordingly. This ensured that participants could safely increase their physical activity while maintaining a diet that supports their overall health goals. The intervention emphasized the importance of consistency and gradual progression to build endurance and foster long-term health benefits.

#### 2.3.3 Walking and dietary regime

As previous recommendations (60, 61), it was strategically designed to incorporate walking, an easily accessible and low-impact form of exercise, with a nutritional plan aimed at enhancing overall health and facilitating weight management. This approach provided a foundation for participants to incorporate physical activity into their daily routines without the need for specialized equipment or training, making it a practical option for a wide range of individuals. Throughout the intervention, participants were encouraged to gradually increase their walking duration and intensity, starting with short walks, and eventually progressing to longer distances as suggested by Varma et al. (61). This gradual increase was carefully monitored to ensure it aligned with each participant’s physical capability and health goals, minimizing the risk of injury while maximizing the health benefits. Simultaneously, a dietary regime tailored to support the increased physical activity was implemented. This regime focused on balanced nutrition, emphasizing whole foods, lean proteins, and healthy fats to fuel the body and aid in recovery. Participants received personalized dietary advice to ensure their nutritional intake supported their walking regimen and overall health objectives.

#### 2.3.4 Exercise with a flexible diet

This approach recognized the proven importance of adaptability in dietary habits, enabling individuals to make choices that fit their preferences and lifestyle while still adhering to nutritional guidelines aimed at supporting their fitness goals and overall health (62, 63). Participants engaged in a variety of exercises tailored to their fitness levels and personal goals, which could include strength training, cardio, and flexibility workouts. The exercise programs were designed based on the suggestions and results of previous research (62) to be scalable and adaptable, ensuring that participants could progress at their own pace and according to their evolving fitness levels. The dietary component of the intervention was equally flexible, emphasizing the importance of a balanced intake of macronutrients while allowing participants to choose foods they enjoyed. This approach aimed to foster a positive relationship with food and exercise, reducing the likelihood of diet fatigue and promoting long-term adherence to healthier lifestyle choices. Regular consultations with fitness and nutritional professionals were an integral part of the intervention, providing participants with ongoing support and guidance. This personalized attention ensured that both the exercise and dietary aspects of the program remained aligned with each participant’s health and wellness objectives, facilitating sustainable lifestyle changes.

#### 2.3.5 Exercises through online programs and applications

This method provided flexibility and convenience, allowing individuals to engage in physical activity at their own pace and on their own schedule. According to the evidence-based online programs (64), the online resources offered a mix of workout types, including strength training, cardio exercises, and flexibility sessions. Interactive features of the applications, such as progress tracking and personalized workout recommendations, fostered a supportive and motivating environment. Participants could adjust their routines based on real-time feedback and evolving fitness goals. The digital platform also facilitated community engagement, where users could share experiences and challenges, creating a sense of camaraderie and accountability. Nutritional guidance complemented the physical activity component, with online resources providing dietary advice, meal planning tools, and tracking functionalities.

### 2.4 Data analysis

For data analysis, Random Forest, and Gradient Boosting Regressors were utilized after implementing feature engineering techniques. These methods were chosen for their efficiency in handling the complexity of the dataset and their ability to predict outcomes effectively. The models’ performance was evaluated based on their accuracy in predicting the long-term effectiveness of the lifestyle interventions. Overall, the study involved two ML tasks:

*Task 1*: A Random Forest (RF) classifier was trained to predict group labels using the change in biometrics over time as input. This step was used to identify the most important features influencing the composite score.

*Task 2*: Regression models (Random Forest Regressor and Gradient Boosting Regressor) were trained to predict the composite score. The labels for this task were the composite scores computed from measurements taken at the end of the two-year study. The input for the regression models consisted of initial biometrics (before the intervention began) and the type of treatment received.

Notably, before proceeding to ML methods, we have ensured that our dataset maintain normality in all measured variables.

The dataset was split into training and validation sets to ensure the robustness and generalizability of the machine learning models. We used a standard split of 80% of the data for training and 20% for validation, which is a common practice in machine learning to balance between learning complexity and validation accuracy. This split allows for adequate learning and model tuning while providing a reliable subset for validating the model’s performance outside of the training dataset.

The decision to use Random Forest Regressor (RFR) and Gradient Boosting Machine (GBM) was based on their suitability for handling complex, non-linear relationships within large datasets with mixed data types. Both models are renowned for their high accuracy, robustness against overfitting, and ability to handle imbalanced datasets, which is particularly pertinent given the variable prevalence rates of different types of eating disorders.

#### 2.4.1 Random forest regressor (RFR)

The RFR is effective due to its ensemble approach, where multiple decision trees aggregate their outcomes to improve predictive accuracy and control over-fitting. Its performance is robust across different types of data distributions, making it ideal for our diverse set of variables.

#### 2.4.2 Gradient boosting machine (GBM)

GBM builds on the concept of boosting weak learners (typically decision trees), focusing on correcting the errors of previous trees in the sequence. This method is particularly effective for complex datasets where patterns can be obscured by noise and where variable interactions are important. GBM’s iterative correction process enhances its ability to fine-tune results and adapt to intricate data nuances.

The composite score was computed to serve as an overall indicator of the effectiveness of the various lifestyle interventions. This score integrates multiple health outcomes relevant to eating disorders, weighted according to their clinical importance as determined by a panel of experts. The following steps outline the computation process:

Selection of variables: Body Fat Percentage (BFP); Waist-Hip Ratio (WHR); Fasting Blood Sugar (FBS); Low-Density Lipoprotein (LDL) Cholesterol; Total Cholesterol (CHO); Weight; Triglycerides (TG).

Normalization: each variable was normalized to a common scale (e.g., *z*-scores) to ensure comparability across different metrics.

Weight Assignment: clinical experts assigned weights to each variable based on their relevance and impact on overall health outcomes for individuals with eating disorders. The weights were determined through a Delphi process involving multiple rounds of consensus-building among the experts.

Aggregation: the normalized and weighted variables were aggregated using a linear combination to form the composite score. The formula used was:

Composite Score = (Weight of Variable 1 * Normalized Value of Variable 1) + (Weight of Variable 2 * Normalized Value of Variable 2) + … + (Weight of Variable *n* * Normalized Value of Variable *n*).

The utilization of composite scores in machine learning analysis is well-established in the literature, particularly in the healthcare domain. Composite scores allow for the integration of multiple health indicators into a single metric, providing a comprehensive measure of overall health outcomes. For instance, Boven et al. (65) demonstrated the use of composite scores in predicting neurodevelopmental outcomes after preterm birth using machine learning models, which enabled the incorporation of diverse health metrics into a unified predictive framework (65). Similarly, Davoudi et al. (66) employed composite scores to ensure fairness in predicting acute postoperative pain, highlighting the importance of combining various health indicators to enhance predictive accuracy and model robustness (66). Furthermore, Killian et al. (67) utilized composite scores to predict health outcomes in pediatric heart transplantation, integrating numerous health factors to form a holistic predictive model (67). These examples illustrate the efficacy of composite scores in enhancing the predictive capabilities of machine learning models by providing a more comprehensive evaluation of health interventions.

**Figure 1:**
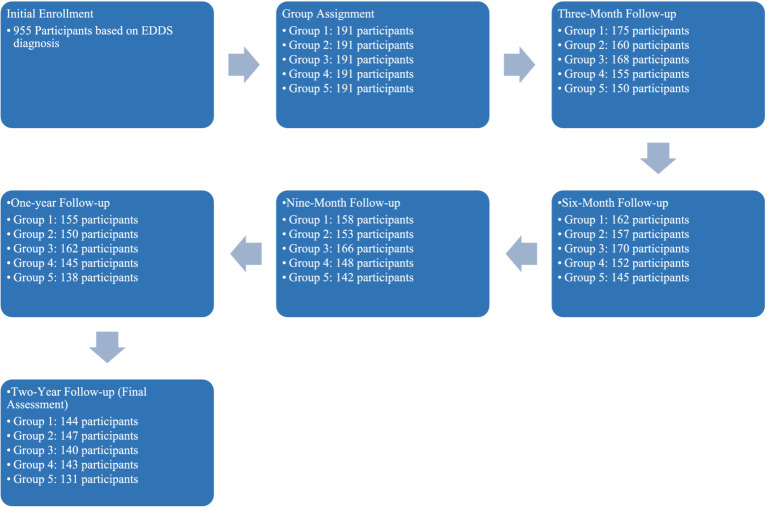
Study process.

After utilizing the Random Forest Classifier to identify feature importance, these weights were adjusted to better reflect the model’s performance. As shown in Figure 1, the feature importance analysis indicated the following weights for the composite score variables: BFP: 0.16, CHO: 0.14, TG: 0.13, LDL: 0.12, Weight: 0.11, FBS: 0.10, and WHR: 0.09. This adjustment ensures that the composite score accurately reflects the most influential health metrics, thereby enhancing the predictive capability of the machine learning models.

## 3 Findings

In our study, the final sample comprised 706 individuals diagnosed with various eating disorders. In terms of age distribution, participants span various age groups, with the largest proportion falling within the 21–30 years bracket (154 participants, 21.8%), followed closely by those aged 11–20 years (92 participants, 13.0%). Gender distribution reveals a slight majority of female participants (461 participants, 65.3%) compared to male participants (245 participants, 34.7%). Educational attainment varies among participants, with the majority holding at least a Bachelor’s degree (283 participants, 40.0%), while smaller proportions hold higher degrees such as a Master’s (90 participants, 12.7%) or Doctorate (33 participants, 4.6%). Regarding annual income, the majority fall into the mid-income bracket ($500–$1,000) (385 participants, 49.6%), followed by low-income earners (<$500) (225 participants, 28.3%), and a smaller proportion in the high-income category (>$1000) (145 participants, 22.1%). Employment status indicates a significant portion of participants being employed full-time (389 participants, 55.1%), with a notable percentage engaged in part-time employment (177 participants, 25.1%), and the remaining participants categorized as unemployed, retired, or homemakers (140 participants, 19.8%).

The inclusion of a broad age range from 11 to 78 years in our study reflects the evolving understanding of eating disorders, which are increasingly recognized across a wider age spectrum. Recent epidemiological studies have shown that while eating disorders are most prevalent among adolescents and young adults, there is a significant and growing incidence in middle-aged and older adults. This trend may be influenced by various factors, including societal pressures, life transitions, and health changes that can trigger or exacerbate eating disorder behaviors (68, 69) (Table 1).

**Table 1:** Demographic characteristics of study participants.

	Characteristic	Frequency (*n*)	Percentage (%)
Age	11–20	92	13.0%
	21–30	154	21.8%
	31–40	125	17.7%
	41–50	105	14.9%
	51–60	80	11.3%
	61+	150	21.3%
Gender	Male	245	34.7%
	Female	461	65.3%
Education level	High School	190	26.9%
	Associate Degree	110	15.6%
	Bachelor’s Degree	283	40.0%
	Master’s Degree	90	12.7%
	Doctorate	33	4.6%
Annual income	Low (<500$)	225	28.3%
	Mid (500$-1000$)	385	49.6%
	High (>1,000$)	145	22.1%
Employment status	Employed Full-time	389	55.1%
	Employed Part-time	177	25.1%
	Unemployed/Retired/Housemaker	140	19.8%

**Figure 2:**
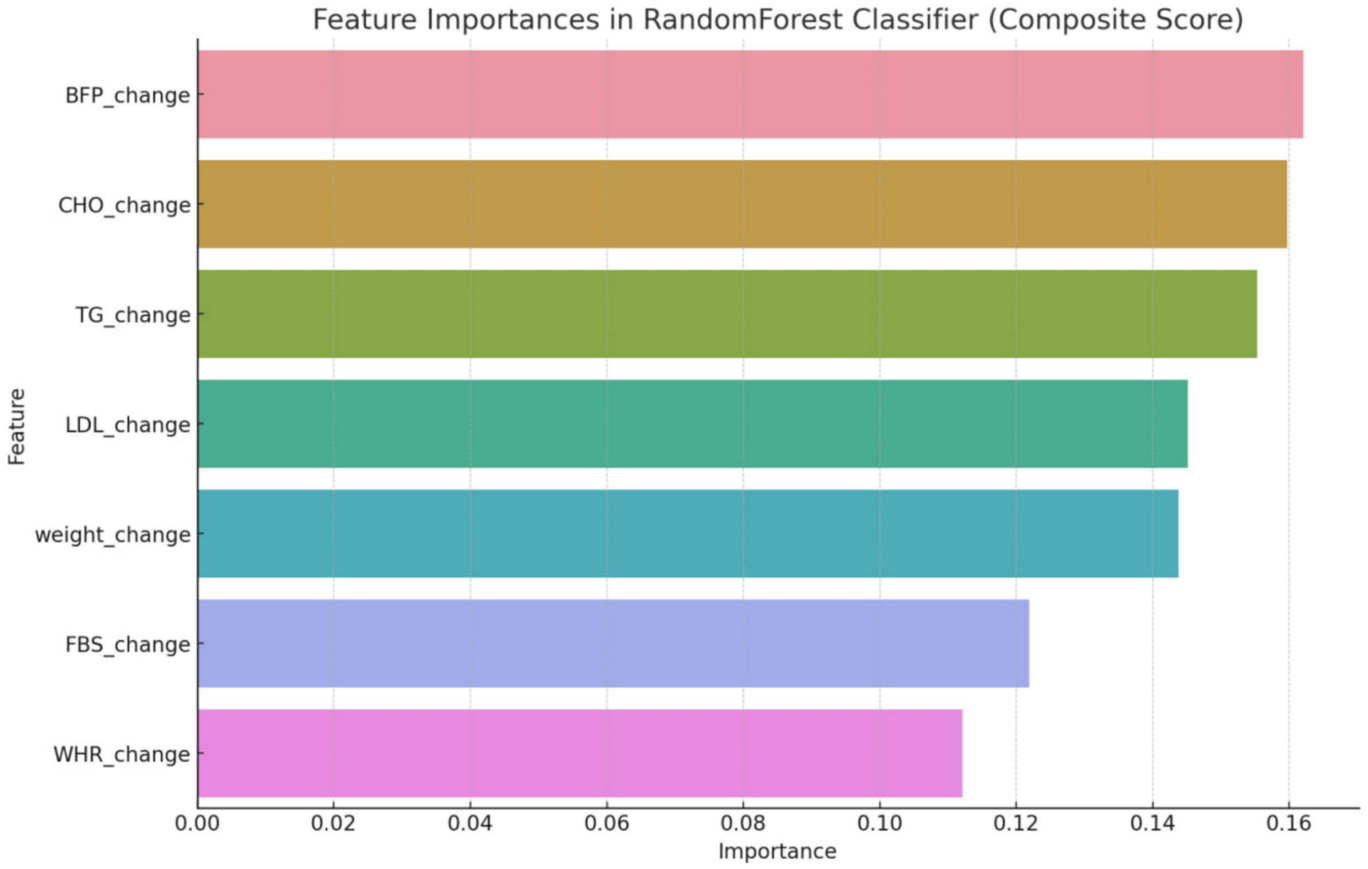
Feature importance by random forest classifier method at initial analysis.

The initial phase of the analysis involved a meticulous feature engineering process. Feature engineering involved creating features that capture the change over time for each variable. This was necessary to account for the dynamic nature of the health outcomes being measured. The use of the Random Forest classifier to discern significant variables helped refine the composite score to ensure it accurately reflected the most influential health metrics. First, it was started with creating features that capture the change over time for each variable and the target included the group labels. Then we utilized a Random Forest classifier to discern which variables significantly influenced the development of a composite score. This score was pivotal in defining the criteria for the subsequent predictive modeling. By focusing on the most influential variables, a more accurate and relevant composite score, indicating overall changes in all variables, was established (Figure 2).

With the composite score defined, the next step entailed training the predictive model using this specifically tailored score. To achieve a comprehensive analysis, two distinct regression models were employed: the Random Forest Regressor and the Gradient Boosting Regressor. These models were chosen for their robustness and efficacy in handling complex, multi-variable datasets, making them ideal for the intricate nature of this study.

**Figure 3:**
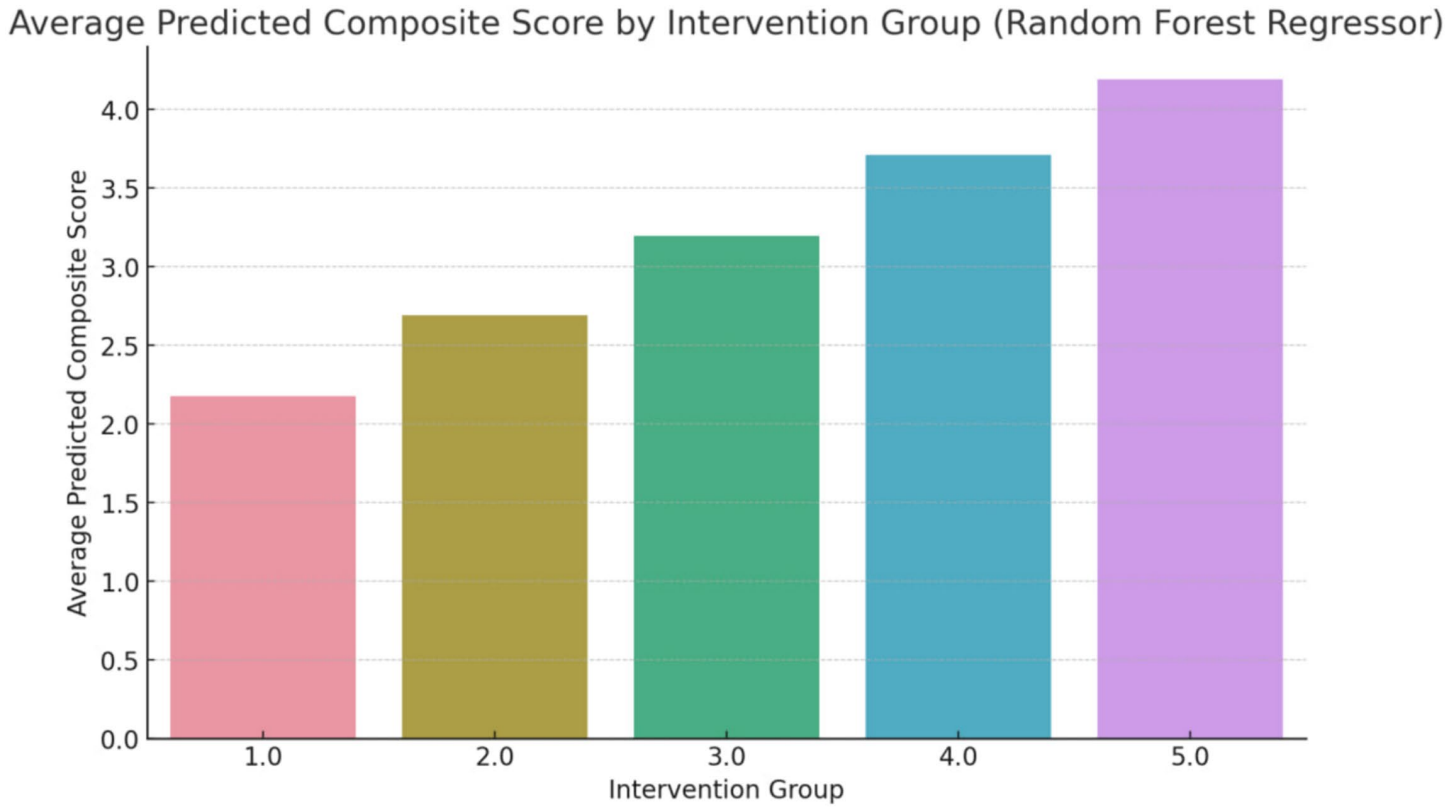
Average predicted composite score (overall changes) using random forest regressor (Group 1: Exercises conducted through online programs and applications; Group 2: Exercise with a flexible diet; Group 3: Walking coupled with dietary regime, Group 4: Aerobic exercises with dietary regime; Group 5: Counseling combined with exercise and dietary regime).

**Figure 4:**
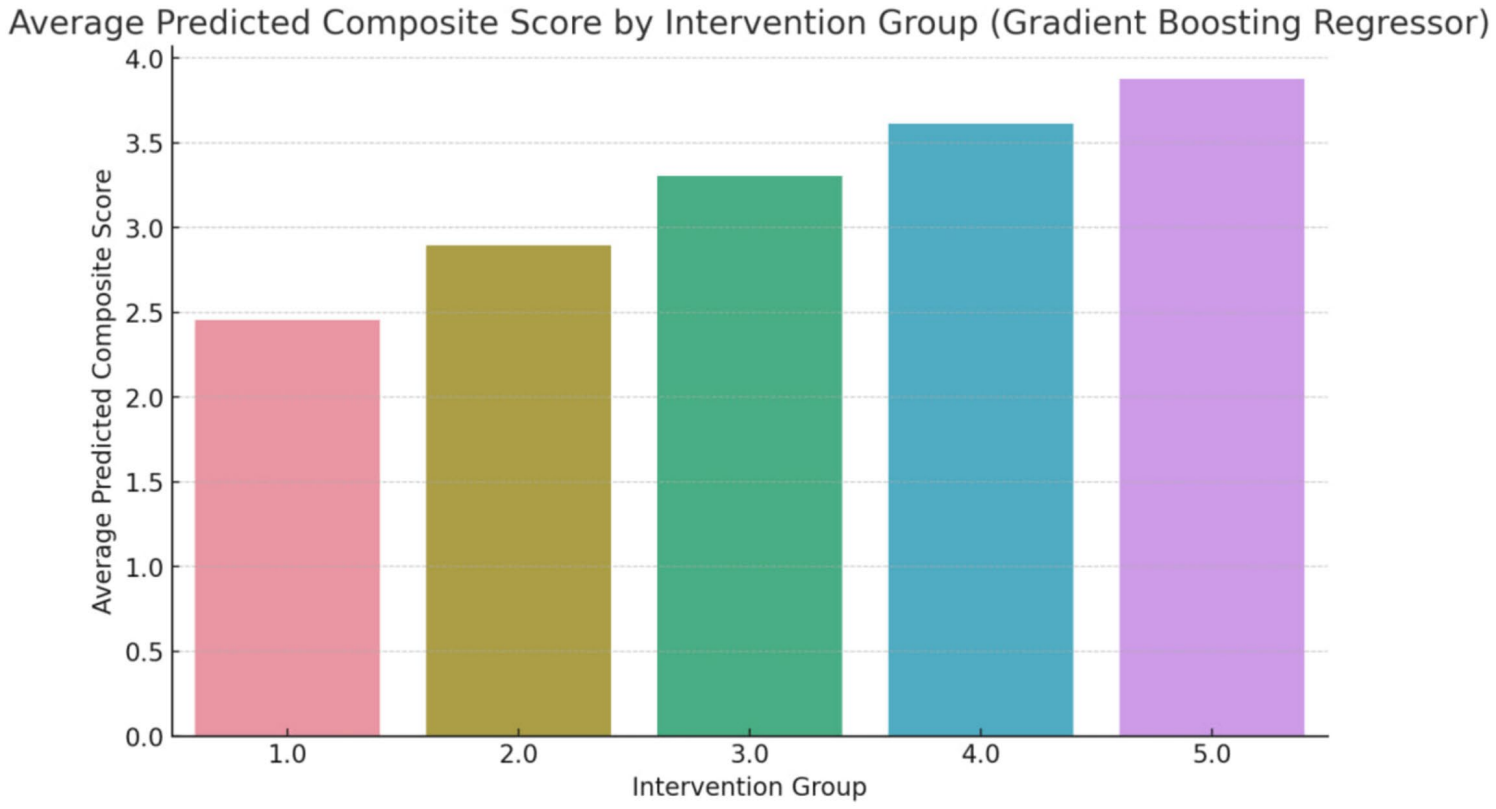
Average predicted composite score (overall changes) using GBM Method.

The composite score, as shown in Figure 3, represents an aggregate measure of the health improvements achieved through each intervention. A higher composite score indicates a more favorable outcome across the selected health metrics. For instance, an average predicted score of 4.3 for Group 5 (Counseling combined with exercise and dietary regime) suggests that this intervention has a significant positive impact on the participants’ health. The score of 4.3 reflects improvements in key health indicators such as reduced body fat percentage, lower LDL cholesterol levels, and better weight management, making this intervention the most effective among those studied (Figure 4).

The below table shows results from an 80–20% split validation approach for two methods: Random Forest Regressor (RFR) and Gradient Boosting Machine (GBM). The RFR method has an accuracy of 85%, precision of 83%, recall of 88%, F1 score of 85%, AUC of 90%, and a Brier score of 0.15. The GBM method shows improved performance with an accuracy of 89%, precision of 87%, recall of 91%, F1 score of 89%, AUC of 93%, and a Brier score of 0.11 (Table 2).

**Table 2:** Performance comparisons of applied methods with selected features.

Results with 80–20% split validation						
Method	Accuracy (%)	Precision (%)	Recall (%)	F1 (%)	AUC (%)	Brier score
RFR	85	83	88	85	90	0.15
GBM	89	87	91	89	93	0.11

**Figure 5:**
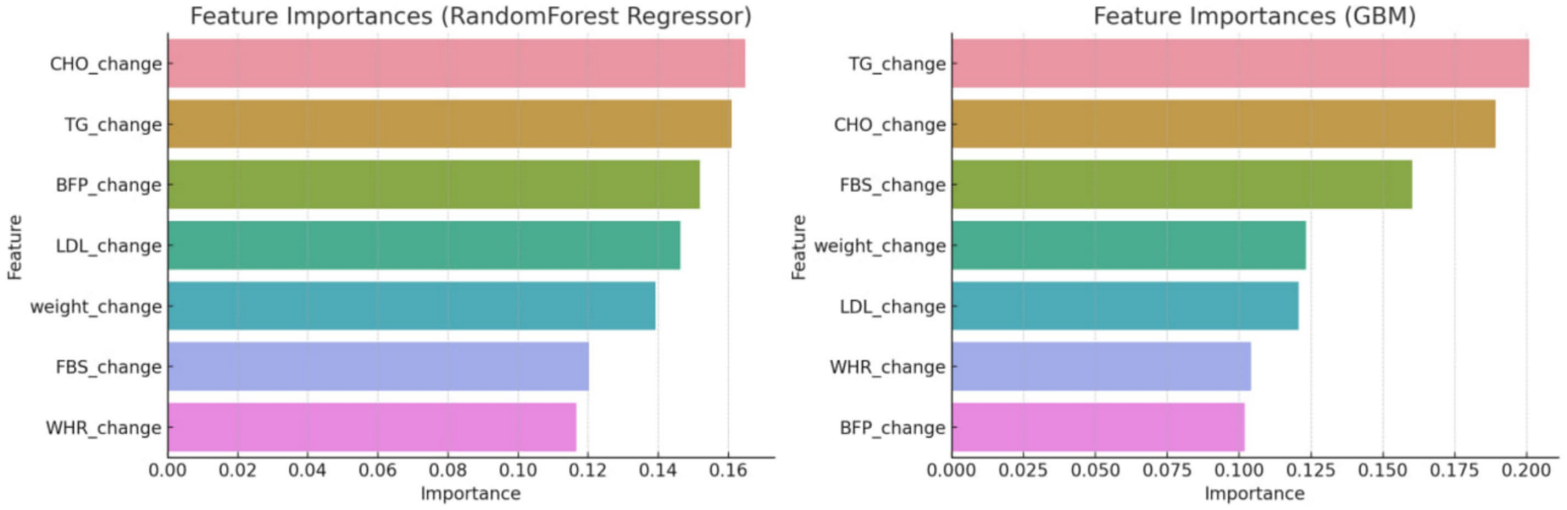
Average predicted composite score (overall changes) using random forest regressor.

To address potential biases arising from unequal group sizes, both oversampling and undersampling techniques were employed, bringing each group to an equal number of 200 subjects. This balanced the dataset to a total of 800 samples, ensuring a fair representation of each intervention group. Subsequently, the composite variable was recalculated for each approach, both for the Random Forest Regressor and the Gradient Boosting Machine (GBM), to maintain consistency and reliability in the models (Figure 5).

**Figure 6:**
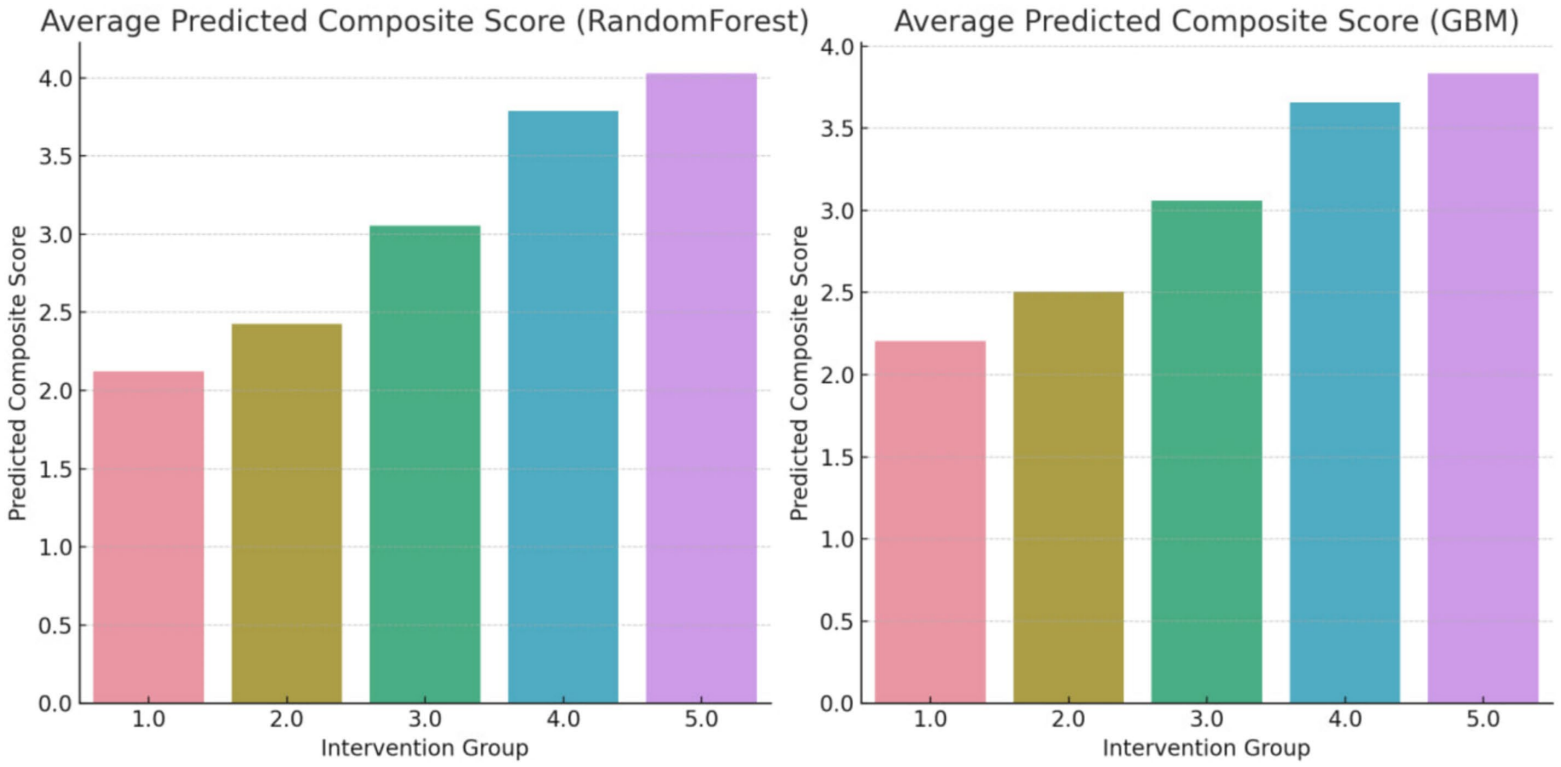
Average predicted composite score (overall changes) using random forest regressor and GBM methods after over and undersampling.

Further feature engineering led to an increase in model accuracy, reaching a notable 90% for both models. Remarkably, the reanalysis with the balanced dataset corroborated the initial findings, reiterating the same ranking order of the interventions. This consistency not only underlines the robustness of the models used but also reinforces the reliability a validity of the results. The consistency in the ranking across different modeling approaches and data configurations highlights the strength of the findings, suggesting a high level of confidence in the predictive capability of the models employed (Figure 6).

The below table presents the performance comparisons after data rebalancing, also using an 80–20% split validation. Post-rebalancing, the RFR method’s metrics slightly improve to an accuracy of 89%, precision of 87%, recall of 91%, F1 score of 89%, AUC of 93%, and a Brier score of 0.10. The GBM method further improves, reaching an accuracy of 90%, precision of 89%, recall of 93%, F1 score of 91%, AUC of 94%, and a Brier score of 0.09. These results indicate that both methods benefit from data rebalancing, with GBM showing a slightly better overall performance (Table 3).

**Table 3:** Performance comparisons of applied methods after rebalancing data with selected features.

Results with 80–20% split validation						
Method	Accuracy (%)	Precision (%)	Recall (%)	F1 (%)	AUC (%)	Brier score
RFR	89	87	91j	89	93	0.10
GBM	90	89	93	91	94	0.09

## 4 Discussion

Lifestyle strategies, as examined in this study, have been central to improving health outcomes and managing various diseases. These interventions, including dietary regimes, exercise programs, counseling, and behavioral therapy, are crucial for encouraging healthy lifestyles and preventing chronic diseases (2). However, a major challenge is assessing their long-term effectiveness, as short-term results do not always mirror their enduring impact on health (6). This study addresses this issue by utilizing machine learning to predict and classify the long-term effectiveness of numerous lifestyle interventions, marking an innovative solution to this ongoing challenge (7).

The interventions were ranked for predicted long-term effectiveness as follows: counseling combined with exercise and dietary regime; aerobic exercises with dietary regime; walking coupled with dietary regime; exercise with a flexible diet; and exercises conducted through online programs and applications. This ranking offers a fresh perspective on the comparative effectiveness of various lifestyle approaches.

In discussing the comparative effectiveness of various lifestyle interventions for individuals with eating disorders, the ranking illuminates the comprehensive benefits of integrating counseling with exercise and dietary regimes. This multifaceted approach addresses not only the physiological aspects of eating disorders but also the critical psychological components, such as depressive and anxiety symptoms, which significantly influence the long-term outcomes and chronicity of these disorders (70). The need for specialized training in handling eating disorders, as emphasized by Levitt (71), underscores the complexity of these conditions and the necessity of a well-rounded understanding and approach by healthcare providers.

The literature review, incorporating studies on dietary regimes and their impact on eating disorders, highlights the diverse factors influencing eating disorder outcomes—from dietary practices to psychological factors like dietary restraint and its relation to treatment outcomes (10, 11). These findings suggest that personalized treatment plans, which consider the unique dietary and psychological needs of everyone, are crucial for effective long-term management and recovery from eating disorders.

The most impactful intervention was the combination of counseling, exercise, and dietary regime. This comprehensive approach is particularly effective as it tackles multiple health and wellness aspects simultaneously. Counseling provides necessary psychological support and motivation, crucial for sustaining lifestyle changes long-term. Including both exercise and dietary changes guarantees an all-encompassing approach to physical health. This is in line with Faulkner et al. (34) and Hannan et al. (36), who showed the effectiveness of combined behavioral change strategies in weight management and emphasized the integration of diet and nutrition counseling in exercise programs for cardiovascular health.

The second most effective intervention was aerobic exercises paired with a dietary regime. Aerobic exercise is known for its cardiovascular benefits, as evidenced by Luo et al. (27), who found a reverse relationship between aerobic exercise capacity and cardiovascular mortality. This intervention’s efficacy is likely boosted by the addition of a dietary regime, as shown by Ho et al. (26) and Smith et al. (25), who reported improvements in neurocognitive functions and cardiovascular risk factors.

Walking combined with a dietary regime was the third most effective. Walking, being a moderate and accessible form of exercise, together with a dietary regime, provides a balanced health improvement method. Its effectiveness is supported by studies like Richardson et al. (24), which linked walking interventions to weight loss, and Griffiths et al. (20), highlighting the importance of structured dietary interventions.

The fourth-ranked intervention, exercise with a flexible diet, underscores the value of personalizing exercise routines and diet flexibility. This approach is particularly suitable for those who prefer variety in their health regimen. The significance of flexibility in exercise programs is emphasized by Ketigian et al. (41) and Silva-Jose et al. (32), advocating adaptable formats to suit individual needs.

Lastly, exercises through online programs, although ranked lowest, still showed substantial effectiveness, particularly where traditional exercise facilities are not available. The growing popularity and benefits of online exercise programs, noted by Kikuchi et al. (30) and Parker et al. (31), indicate their role in promoting physical activity and well-being, especially in isolated or restricted settings.

The use of machine learning in predicting health outcomes is a cornerstone of this study. Machine learning models have been successfully applied in various healthcare areas, from predicting obesity-related risks (72) to outcomes in critical illnesses (73). The incorporation of machine learning in this study aligns with the trend of applying advanced analytical techniques in healthcare (74, 75).

This research significantly contributes to our understanding of the long-term effectiveness of diverse lifestyle interventions. By integrating machine learning, it offers a novel approach to evaluating these interventions, providing valuable insights for healthcare professionals, policymakers, and individuals. This study not only adds to the existing literature on lifestyle interventions but also demonstrates the promising use of machine learning in predicting health outcomes, paving the way for more personalized and impactful health strategies.

### 4.1 Limitations

The participant pool, drawn from a single geographic location and primarily consisting of a specific demographic, limits the generalizability of the findings. Additionally, the interventions’ long-term effects were predicted rather than directly observed, which could affect the accuracy of outcomes. Furthermore, psychological, and social adherence factors were not included in the model, potentially overlooking critical aspects influencing the success of lifestyle interventions. It is important to acknowledge that the composite score, while based on clinical expert opinion and adjusted through feature importance analysis, may not capture all aspects of treatment effectiveness. Factors such as psychological well-being and social adherence, which are crucial for comprehensive assessment, were not included. This gap represents a potential limitation of the study. The term ‘long-term effects’ refers to outcomes observed after 2 years, which is the duration of the study. While this period addresses the interventions’ effectiveness, it does not fully encompass longer-term outcomes beyond 2 years.

### 4.2 Future research directions

Future research should aim to broaden the demographic and geographic scope of participants to enhance the generalizability of results. It would be beneficial to incorporate longitudinal studies that directly observe the long-term effects of interventions, rather than relying solely on predictions. Expanding the variables to include psychological and social adherence factors could offer a more holistic understanding of intervention success. Additionally, exploring advanced machine learning techniques and integrating genomics-driven approaches may uncover deeper insights into personalized health strategies.

### 4.3 Suggestions for practice and implications

The findings underscore the importance of personalized health strategies in managing eating disorders through lifestyle interventions. Healthcare providers should consider integrating counseling with exercise and dietary regimes, as this combination was found most effective. Policymakers could leverage these insights to allocate resources toward interventions demonstrating higher efficacy. There is a potential for machine learning models to play a pivotal role in predictive healthcare, guiding the development of targeted intervention strategies. Emphasizing a multifaceted approach that includes psychological support alongside physical health interventions could significantly enhance patient outcomes. This study’s approach highlights the potential for data-driven, personalized healthcare solutions to address complex health conditions, suggesting a shift toward more adaptive and responsive healthcare planning.

## Data availability statement

The raw data supporting the conclusions of this article will be made available by the authors, without undue reservation.

## Ethics statement

All research included in this review was evaluated for adherence to ethical standards, particularly regarding participant consent and data confidentiality. This study has been registered with the Institutional Ethics Board of KMAN Publication Institute, under the registration code KEC.2021.8A1, ensuring compliance with all ethical standards and protocols for research. The studies were conducted in accordance with the local legislation and institutional requirements. The participants provided their written informed consent to participate in this study.

## Author contributions

KI: Conceptualization, Writing – original draft. KP: Writing – review & editing. AE: Writing – review & editing. GZ: Writing – review & editing. BK: Writing – review & editing. TR: Writing – review & editing. KW: Writing – review & editing. MT: Conceptualization, Writing – review & editing.
